# Fully connecting the Observational Health Data Science and Informatics (OHDSI) initiative with the world of linked open data

**DOI:** 10.5808/GI.2019.17.2.e13

**Published:** 2019-06-11

**Authors:** Juan M. Banda

**Affiliations:** Panacea Laboratory, Department of Computer Science, Georgia State University, Atlanta, GA 30303, USA

**Keywords:** clinical informatics, common data model, controlled vocabularies, linked open data, RDF, semantic web

## Abstract

The usage of controlled biomedical vocabularies is the cornerstone that enables seamless interoperability when using a common data model across multiple data sites. The Observational Health Data Science and Informatics (OHDSI) initiative combines over 100 controlled vocabularies into its own. However, the OHDSI vocabulary is limited in the sense that it combines multiple terminologies and does not provide a direct way to link them outside of their own self-contained scope. This issue makes the tasks of enriching feature sets by using external resources extremely difficult. In order to address these shortcomings, we have created a linked data version of the OHDSI vocabulary, connecting it with already established linked resources like bioportal, bio2rdf, etc. with the ultimate purpose of enabling the interoperability of resources previously foreign to the OHDSI universe.

**Availability:** The resource described in this article is available in two different ways: fully constructed RDF graph (GRAPHcompressed files), https://github.com/thepanacealab/OHDSI2RDF; scripts to generate RDF graph, https://github.com/thepanacealab/OHDSI2RDF.

## Introduction

The Observational Health Data Science and Informatics (OHDSI) is a world-wide initiative, which over the course of five years has managed to bring groups of researchers all over the world together in converting their clinical patient data (electronic health records, claims, clinical registries) into the Observational Medical Outcomes Partnership (OMOP) common data model (CDM). This initiative has built a large set of publicly available tools which allow researchers to standardize the way they build patient cohorts, characterize their data [1], perform large scale patient level prediction studies [2], and perform electronic phenotyping [3]. In just a few years the OHDSI initiative has managed to perform large-scale studies involving over 200 million patients [4], answer drug safety questions by analyzing the association of the anticonvulsant levetiracetam with increased risk for angioedema in 10 international databases [5], and has characterized the effectiveness of second-line treatment of type 2 diabetes after initial therapy with metformin in over 246 million patients [6]. All of these massive studies have been made possible thanks to the use of a CDM and a standardized vocabulary. This strength becomes a weakness as the vocabulary standardizes multiple external vocabularies, ontologies and term sets, such as SNOMED, RxNorm, MeSH, and 90+ others, but it does not provide an easy way to link them to additional resources such as the Unified Medical Language System (UMLS) [7] and other linked open data resources like Bio2rdf [8] and BioPortal [9]. During our time at the Biomedical Link Data Hackathon 5 in Kashiwa, Japan we developed the first attempt to create an RDF version of the OHDSI vocabulary with linkages to UMLS and BioPortal.

## Methods

In order to link the OHDSI vocabulary with UMLS, we will leverage Ananke [10], a resource built for the mapping of UMLS Concept Unique Identifiers (CUIs) into OHDSI concept_id’s, which are the unique identifiers assigned to all concepts in the vocabulary. This will allow us to use BioPortals URI’s for the CUIs and make the necessary connections when using their SPARQL endpoints for federated queries. All other Python 2.7 code just iterates through the vocabulary concepts, find proper UMLS matches and writes out each entry using a predefined schema. The conversion process assumes the OHDSI vocabulary files are in the same folder, as well as the Ananke mappings. If the researcher does not have a full copy of the OHDSI vocabulary, we provide an already built RDF graph for Vocabulary version v5.0 11-FEB-19.

## Results and Discussion

The RDF conversion results in a total of 24 million triples and takes around 15 minutes. Our resource links a total of 861,732 OHDSI concept_id's from SNOMED, 286,256 concept_id's from RxNORM, 109,706 concept_id's from ICD10, and 22,029 concept_id's from ICD9, all linked directly to bioportal. We also include 1,321,986 mappings to UMLS via Ananke [10].

Our initial goals for this resource were to bring into the OHDSI context semantic enrichment of longitudinal clinical study data, as it has been shown to be quite effective in the past [11,12]. Our particular practical application of interest is taking advantage of the resource for electronic phenotyping purposes. As authors of the Automated PHenotype Routine for Observational Definition, Identification, Training and Evaluation (APHRODITE) R package [3], our goals were as follows.

**Fig. 1.**
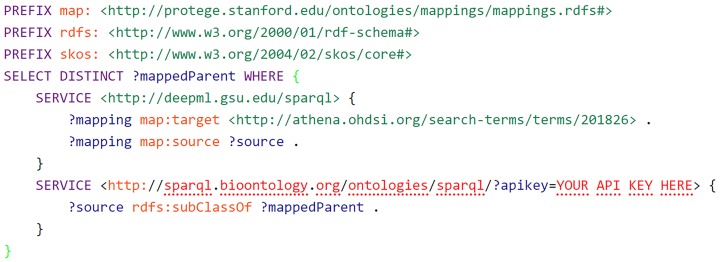
Sample federated SPARQL query to retrieve parent elements for a specific SNOMED concept.

(1) Be able to expand and enrich our feature sets for phenotyping. With one of the main feature spaces of APHRODITE being clinical narratives, these are annotated using the OHDSI vocabulary. Having a linked version of it will allow us to expand any particular feature domain with other linked resources to SNOMEDCT, RxNORM, etc. Fig. 1 shows a sample query were we expand the SNOMED concept for “Type 2 diabetes mellitus” with all its available parents in BioPortal via a federated query.

(2) One of the outputs of APHRODITE, besides a machine learning model for the target phenotype, is a list of relevant features that add interpretability to any model. This list of features covers the most important domains in the OHDSI CDM and vocabulary. We want to be able to produce this list as a linked resource that will allow researchers to enhance their understanding by being able to semantically link them to other resources like the Human Phenotype Ontology [13] among others.

We believe that such interoperability will enable other researchers to generate enhanced evidence by linking outside of the OHDSI CDM and vocabulary with additional resources available, such as phenotype annotations from PubMed abstracts automatically [14], provide extra context for word embeddings models built from clinical narratives [15], which in theory can help the embeddings be more specific by providing additional context [16], and many additional applications. This resource brings us one step closer to enrich EHR, claims, and registry patient data with the world of linked open data.
